# Clinical predictors of renal non-recovery in acute respiratory distress syndrome

**DOI:** 10.1186/s12882-019-1439-2

**Published:** 2019-07-10

**Authors:** Anupol Panitchote, Omar Mehkri, Andrei Hasting, Tarik Hanane, Sevag Demirjian, Heather Torbic, Eduardo Mireles-Cabodevila, Sudhir Krishnan, Abhijit Duggal

**Affiliations:** 1 Department of Critical Care, Respiratory Institute, Cleveland Clinic, Cleveland, OH USA; 2 Division of Critical Care Medicine, Department of Medicine, Faculty of Medicine, Khon Kaen University, Khon Kaen, Thailand; 3 Department of Nephrology, Cleveland Clinic, Cleveland, OH USA; 4 Department of Pharmacology, Cleveland Clinic, Cleveland, OH USA

**Keywords:** Acute kidney injury, Acute respiratory distress syndrome, Renal recovery, Mechanical ventilation, Septic shock, Predictor

## Abstract

**Background:**

Acute kidney injury (AKI) is the most common extra-pulmonary organ failure in acute respiratory distress syndrome (ARDS). Renal recovery after AKI is determined by several factors. The objective of this study was to determine the predictors of renal non-recovery in ARDS patients.

**Methods:**

A single center retrospective cohort study of patients with AKI after onset of ARDS. Patients with preexisting chronic kidney disease or intensive care unit stay < 24 h were excluded. AKI staging was defined according to the Kidney Disease Improving Global Outcomes (KDIGO) 2012 guidelines. Renal non-recovery was defined as death, dialysis dependence, serum creatinine ≥1.5 times the baseline, or urine output < 0.5 mL/kg/h more than 6 h.

**Results:**

Of the 244 patients that met study criteria, 60 (24.6%) had stage I AKI, 66 (27%) had stage II AKI, and 118 (48.4%) had stage III AKI. Of those, 148 (60.7%) patients had renal non-recovery. On multivariable analysis, factors associated with renal non-recovery were a higher stage of AKI (odds ratio [OR] stage II 5.71, 95% confidence interval [CI] 2.17–14.98; OR stage III 45.85, 95% CI 16.27–129.2), delay in the onset of AKI (OR 1.12, 95% CI 1.03–1.21), history of malignancy (OR 4.02, 95% CI 1.59–10.15), septic shock (OR 3.2, 95% CI 1.52–6.76), and a higher tidal volume on day 1–3 of ARDS (OR 1.41, 95% CI 1.05–1.90). Subgroup analysis of survival at day 28 of ARDS also found that higher severity of AKI (OR stage II 8.17, 95% CI 0.84–79.91; OR stage III 111.67, 95% CI 12.69–982.91), delayed onset of AKI (OR 1.12, 95% CI 1.02–1.23), and active malignancy (OR 6.55, 95% CI 1.34–32.04) were significant predictors of renal non-recovery.

**Conclusions:**

A higher stage of AKI, delayed onset of AKI, a history of malignancy, septic shock, and a higher tidal volume on day 1–3 of ARDS predicted renal non-recovery in ARDS patients. Among survivors, a higher stage of AKI, delayed onset of AKI, and a history of malignancy were associated with renal non-recovery.

**Electronic supplementary material:**

The online version of this article (10.1186/s12882-019-1439-2) contains supplementary material, which is available to authorized users.

## Background

Over the past 15 years, the definition of acute kidney injury (AKI) has evolved. These changes in definitions have facilitated an increase in diagnosing AKI [1]. AKI is prevalent in critical illness, and one third of critically ill patients develop AKI during the course of their intensive care unit (ICU) admission [2]. Similarly, approximately 40% of acute respiratory distress syndrome (ARDS) patients develop AKI [3]. Most develop AKI in the first few days after the onset of ARDS [3]. But AKI recovery can take distinctive trajectories based on the severity of the initial insult [4].

A reduction of serum creatinine (SCr), improvement of estimated glomerular filtration rate (eGFR), and a need for dialysis have been used frequently to assess renal recovery [4]. Acute Disease Quality Initiative has also established a renal recovery definition [5]. These definitions are diverse and their application in critically ill patients can be variable. Recently, Kellum et al. recently categorized renal recovery into 5 phenotypic groups in critically ill patients. These included early and late sustained reversal, relapsing AKI with and without complete renal recovery, and never-reversed AKI [6]. This definition has significant clinical application as it helps with appropriate resource utilization for critically ill patients with AKI.

In both hospitalized and critically ill patients, several factors including underlying co-morbidities, initial severity of the acute illness, and severity of AKI have been associated with renal recovery [4]. In these populations the development of other organ failures as well as complex cardiopulmonary interactions can have significant impact on the rates of renal recovery [7]. AKI is associated with worse outcomes in ARDS [3]. Recognition of potentially modifiable risk factors can be instrumental in enhancing the likelihood of renal recovery [5].

The main objective was to investigate the predictors of renal non-recovery in ARDS patients. We also examined the patterns of AKI reversal and assessed the time course of AKI recovery.

## Methods

We conducted a retrospective cohort study from January 1, 2010, to May 31, 2017. Inclusion criteria were adult (> 18 years old) patients admitted to a medical ICU with a diagnosis of ARDS based on the Berlin definition [8] and AKI based on the Kidney Disease Improving Global Outcomes (KDIGO) 2012 guidelines. We excluded patients with preexisting chronic kidney disease (CKD) stage 3a to stage 5 based on GFR category (eGFR < 60 mL/min/1.73m^2^) [9], AKI prior to the onset of ARDS, or ICU stay < 24 h. This study was approved by the Cleveland Clinic Institutional Review Board (#17–806) and granted a waiver of informed consent.

### Data collection and definition of AKI and renal recovery

AKI and AKI severity were defined according to KDIGO 2012 guidelines [10] using SCr and urine output criteria. Baseline SCr values were assessed using the mean value between 7 and 365 days before hospitalization. Patients where baseline renal function was not available, the baseline SCr was imputed by using the Modification of Diet in Renal Disease (MDRD) equation for a normal GFR of 75 mL/min per 1.73 m^2^ [11].

Renal recovery was defined based on work by Kellum et al. [6] and Acute Disease Quality Initiative (ADQI) 16 Workgroup [5] (1) rapid sustained reversal: recovery from AKI within 48 h, (2) late sustained reversal: reversal after 48 h and sustained through 28 days after AKI diagnosis or hospital discharge, (3) relapsing AKI with complete recovery, (4) relapsing AKI without complete recovery and (5) never recovery. The first three categories were classified as complete renal recovery while the last two categories were classified as renal non-recovery. Patients with AKI were followed up to 28 days after AKI diagnosis or till hospital discharge. Sustained AKI reversal was defined as achieving renal recovery for more than 48 h. AKI after 48 h of reversal was considered as a new AKI episode [5]. Complete renal recovery was defined as alive, free of RRT, improvement of SCr < 1.5 times the baseline SCr, and urine output > 0.5 mL/kg/hour more than 6 h [12].

Collected data were extracted from electronic medical records. Day 1 was defined as the first day that patient met criteria of ARDS, irrespective of ICU admission date [13]. Demographic data that were recorded included: age, sex, ethnicity, race, height, body mass index (BMI), comorbidities, Charlson comorbidity index, ARDS risk factors, and echocardiographic findings. Severity of illness including the Sequential Organ Failure Assessment (SOFA) score and the Acute Physiology, Age, Chronic Health Evaluation (APACHE) III score were recorded on day 1 of ARDS. For outside transfers SOFA and APACHE III score were recorded in the first 24 h of hospital admission. Mechanical ventilation parameters, arterial blood gas, serum lactate, intake, output, and percentage of fluid overload were collected for the first three days, day 7, and day 14 of onset of ARDS. Percentage of fluid overload was calculated using the following formula [14]: Percentage of fluid overload (%) = [fluid intake (L) – total output (L)] / body weight at ICU admission (kg.) × 100. Serum creatinine, urine volume, and use of renal replacement therapy (RRT) were recorded until 28 days after ARDS diagnosis or hospital discharge in order to determine the highest stage of AKI. For patients with ARDS who developed AKI, SCr, urine volume, and use of RRT were recorded until 28 days after AKI diagnosis or hospital discharge in order to determine renal recovery. The onset of AKI was classified into: early onset (within 2 days after ARDS diagnosis) and late onset (after 2 days of ARDS diagnosis) [15]. Diuretic use was recorded during day 2 to 7 of ARDS. Exposure to nephrotoxic agents (including: antimicrobial nephrotoxic agents [vancomycin, aminoglycoside, sulfamethoxazole-trimethoprim, colistin, amphotericin B], contrast agents, angiotensin converting enzyme inhibitors, calcineurin inhibitors, and non-steroid anti-inflammatory drugs), septic shock and vasopressor use were recorded daily until day 28 of ARDS. Septic shock was defined according to the Sepsis-3 consensus definition [16]. The primary outcomes were factors associated with renal non-recovery. Study data was collected and managed using REDCap [17].

### Statistical analysis

Continuous variables were presented as mean, standard deviation, median, interquartile range (IQR) as appropriate. Categorical variables were described as counts and percentages. The study group was divided into two groups (complete renal recovery and renal non-recovery). Student’s t-test or Wilcoxon rank sum test was used to compare continuous variables. Chi-square test or Fisher’s exact test was used for categorical variables. Missing data of all cohort patients and sub-group patients (alive at day 28 of ARDS) were handled using multiple imputation by chained equations and analyzed 50 imputed data sets in order to complete logistic regression. The imputation process included variables that were incorporated into both regression model and also included outcomes variables [18]. Calculation of missing values were done in R version 3.5.1 using automatic predictor selection tool of the mice 3.0.0 package [19]. The procedure assumed the missing data to be missing at random. The model estimates and standard errors from each data sets were combined into a single set of results using Rubin’s rules.

A binary logistic regression model was used for analyzing the factors associated with renal non-recovery. Model selections used backward and forward stepwise approach. To build a multivariable regression model, univariable regression was first performed. The variables significant at *p* < 0.1 on univariable analysis were identified as potential predictor variables and entered into a multivariable regression model. Area under the receiver operating characteristic were calculated for determination the model performances. Since RRT was highly correlated with severity of AKI, variable of RRT was not selected into the multivariable model. However, we separately analyzed the effect of RRT to renal recovery in subgroup patients with stage III AKI. All the statistical analyses were performed with R (version 3.5.1). The level of statistical significance was set at *p* < 0.05 (two tailed).

### Sensitivity analysis

Multivariable analyses of variables associated with renal non-recovery were performed with and without data imputation. Non-imputed data was analyzed using a binary logistic regression. Backward stepwise approach was used for model selection. Several models were compared using likelihood ratio tests.

## Results

**Fig. 1:**
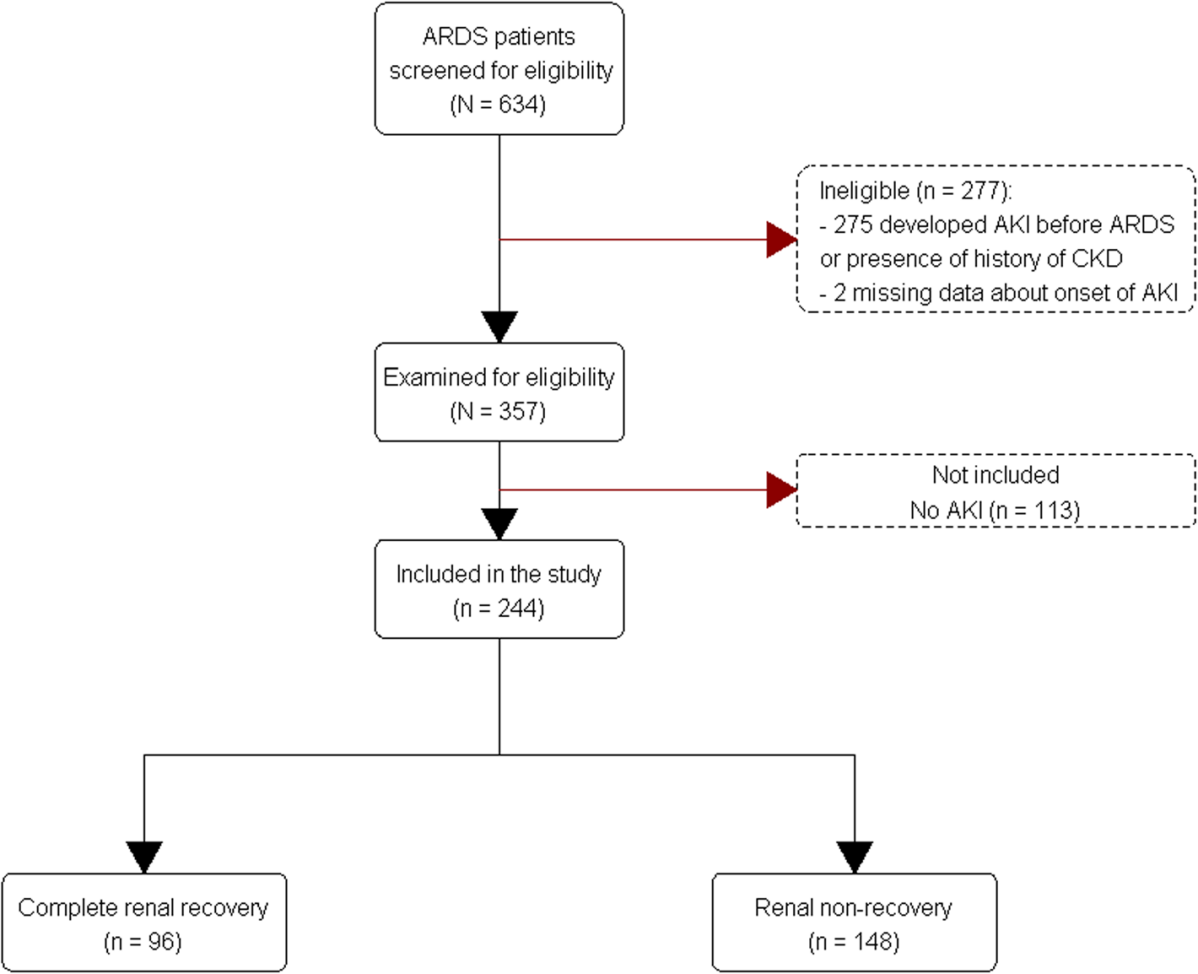
Flow diagram of the study population

A total of 634 ARDS patients were screened. The 357 patients were examined for eligibility; however, 113 patients did not develop AKI until day 28 of ARDS (Fig. 1). We included 244 patients with AKI in the study, 60 (24.6%) patients had stage I AKI, 66 (27%) patients had stage II AKI, and 118 (48.4%) patients had stage III AKI. Of those, 207 (84.8%) patients were diagnosed AKI based on Scr criteria, while 37 (15.2%) patients were diagnosed AKI based on urine output criteria. Among patients with AKI, 148 (60.7%) patients did not have complete renal recovery, while 96 (39.3%) patients had complete renal recovery at day 28 after AKI or at hospital discharge. In patients with complete renal recovery, rapid sustained reversal was seen in 32 (33.3%), late sustained reversal in 45 (46.9%), and relapsing AKI with subsequent complete recovery in 19 (19.8%). In patients without complete recovery, 14 (9.5%) relapsed without subsequent recovery, and 134 (90.5%) never recovered at any point.

The percentage of missing data across the 14 potential variables that were put in the full regression model varied between 0 and 44.7%. The percentage of fluid overload on day 7 and average tidal volume on day 1–3 were the two most common missing variables, 44.7 and 7.4%, retrospectively (Additional file 1: Table S1).

Baseline characteristics between patients with and without complete renal recovery are shown in Table 1. The renal non-recovery patients had a significantly higher severity of illness, active malignancy (31.8% vs 12.5%, *p* < 0.001), septic shock (70.3% vs 39.6%, *p* < 0.001). The time to development of the highest stage of AKI from ARDS onset was longer than in non-recovery patients (4 [2.7] vs 3 [2–6], *p* = 0.02) (Table 1). The non-recovery patients received higher average tidal volume on day 1–3 (7.6 [6.9–8.6] vs 7.3 [6.6–8.0], *p* = 0.01), neuromuscular blocking agents (72 [48.6%] vs 27 [28.1%], *p* = 0.001), inhaled vasodilators (46 [31.1%] vs 18 [18.8%], *p* = 0.03), and recruitment maneuvers (17 [11.5%] vs 3 [3.1%], *p* = 0.02). In addition, the non-recovery patients had a higher percentage of fluid overload on day 7 of ARDS (10 [8.7] vs 5.5 [8.2], *p* = 0.003) while receiving a lower proportion of furosemide on day 2–7 of ARDS (71 [48%] vs 63 [65.6%], *p* = 0.01) (Table 2).

**Table 1:** Baseline characteristics of all 244 patients by renal recovery

Characteristic	Recovery (96)	Non-recovery (148)	*p* value
Age, median (IQR), years	56 (44–66)	56 (43.8–64)	0.57
Male sex, n (%)	55 (57.3)	82 (55.4)	0.77
Body mass index, median (IQR), kg/m^2^	30.6 (24.5–37.2)	30.2 (25.2–39.4)	0.54
Race, n (%)			
White	70 (72.9)	100 (67.6)	0.37
Black or African American	25 (26)	33 (22.3)	0.50
SOFA, mean (SD), points	10.6 (3)	12.7 (3.6)	< 0.001^*^
Non-renal SOFA, mean (SD), points	9.8 (2.7)	11.3 (3)	< 0.001^*^
APACHE III, mean (SD), points	105 (29)	121 (31)	< 0.001^*^
Charlson comorbidities index, median (IQR), points	3 (1–5)	3 (1–5)	0.61
Severity of ARDS on day 1			
Mild	13 (15.3)	20 (15.2)	0.22
Moderate	41 (48.2)	49 (37.1)	
Severe	31 (36.5)	63 (47.7)	
Comorbidities, n (%)			
Chronic lung diseases	34 (35.4)	40 (27)	0.16
Diabetes	25 (26)	47 (31.8)	0.34
Active malignancies	12 (12.5)	47 (31.8)	< 0.001^*^
Liver disease	7 (7.3)	21 (14.2)	0.10
Heart failure	14 (14.6)	8 (5.4)	0.01^*^
Recent surgery within 3 months.	4 (4.2)	6 (4.1)	1.00
Cause of ARDS, n (%)			
Pneumonia	82 (85.4)	122 (82.4)	0.54
Aspiration	20 (20.8)	23 (15.5)	0.29
Non-pulmonary sepsis	6 (6.2)	15 (10.1)	0.29
Pancreatitis	5 (5.2)	3 (2)	0.27
Echocardiographic findings			
Ejection fraction, median (IQR),%	58.5 (55–64)	60 (55–65)	0.74
RVSP, median (IQR), mm Hg	39 (32.5–49)	40.5 (31.3–50.8)	0.86
Vasopressors use	66 (68.8)	136 (91.9)	< 0.001^*^
Septic shock	38 (39.6)	104 (70.3)	< 0.001^*^
Nephrotoxic agents	93 (96.9)	142 (95.9)	1.00
Median time to develop AKI	3 (2–6)	4 (2–7)	0.02^*^
RRT initiation from highest AKI onset	0 (0–0)	0 (0–2)	0.18
Known baseline SCr, n (%)	56 (58.3)	89 (60.1)	0.78
Baseline SCr, mean (SD), mg/dL	0.85 (0.22)	0.8 (0.18)	0.18
eGFR, median (IQR), mL/min per 1.73 m^2^	92.5 (73.2–109.5)	97.1 (82.2–112.5)	0.10

*AKI* = acute kidney injury, *APACHE* = acute physiology, age, chronic health evaluation, *ARDS* = acute respiratory distress syndrome, *eGFR* = estimated glomerular filtration rate, *IQR* = interquartile range, *RRT* = renal replacement therapy, *RVSP* = right ventricular systolic pressure, *SCr* = serum creatinine, *SD* = standard deviation, *SOFA* = sequential organ failure assessment

^*^p < 0.05 when compared with patients with complete renal recovery

**Table 2:** Ventilator settings, arterial blood gases averaged on day 1 to 3 and other therapies

Ventilator settings	Recovery (96)	Non-recovery (148)	*p* value
Spontaneous tidal volume, median (IQR), mL	474 (417–539)	499 (428–555)	0.13
Tidal volume, median (IQR), (mL/kg PBW)	7.3 (6.6–8)	7.6 (6.9–8.6)	0.01^*^
PEEP, median (IQR), cm H_2_O	10 (8–13)	11 (8.4–14)	0.08
FiO_2_, median (IQR)	0.7 (0.53–0.82)	0.7 (0.58–0.9)	0.24
Plateau pressure, median (IQR), cm H_2_O	26.5 (21.3–34.4)	27.5 (23–33)	0.45
Plateau pressure > 30 cm H_2_O, n (%)	22 (37.9)	29 (35.4)	0.76
Driving pressure, median (IQR), cm H_2_O	15.0 (11.5–19)	14.5 (12–19)	0.85
Mean airway pressure, median (IQR), cm H_2_O	17.7 (14.5–21.8)	18 (14.8–21.2)	0.82
Peak airway pressure, mean (SD), cm H_2_O	31 (6.9)	31.3 (7.6)	0.82
Minute ventilation, median (IQR), L/min	11 (9.2–12.9)	11.6 (9.9–13.5)	0.20
Arterial blood gas			
Arterial pH, median (IQR)	7.36 (7.3–7.41)	7.35 (7.29–7.39)	0.06
PaCO_2_, median (IQR), mm Hg	43.7 (38–51.2)	41.3 (34.8–48)	0.07
PaO_2_, median (IQR), mm Hg	87.7 (74.8–112)	87.3 (76–109.2)	0.93
PaO_2_:FiO_2_, median (IQR)	138 (111–178)	138 (95–195)	0.94
Oxygenation index, median (IQR)	15.6 (9.2–23.5)	13.9 (9.1–24.4)	0.89
Rescue therapies, n (%)			
Continuous neuromuscular blocking agents	27 (28.1)	72 (48.6)	0.001^*^
Inhaled vasodilators	18 (18.8)	46 (31.1)	0.03^*^
Prone positioning	14 (14.6)	20 (13.5)	0.81
Extracorporeal membrane oxygenation	3 (3.1)	4 (2.7)	1.00
Recruitment maneuvers	3 (3.1)	17 (11.5)	0.02^*^
High frequency oscillatory ventilation	2 (2.1)	9 (6.1)	0.21
Other therapies			
Sedative drugs, n (%)	77 (80.2)	117 (79.1)	0.83
Analgesic drugs, n (%)	70 (72.9)	106 (71.6)	0.83
Antipsychotic drugs, n (%)	48 (50)	56 (37.8)	0.06
Furosemide on day 2–7, n (%)	63 (65.6)	71 (48)	0.01^*^
Fluid overload on day 7, mean (SD), %	5.5 (8.2)	10 (8.7)	0.003^*^

*FiO*_*2*_ = fraction of inspired oxygen, *IQR* = interquartile range, *PaCO*_*2*_ = partial pressure of carbon dioxide in arterial blood, *PaO*_*2* =_ partial pressure of oxygen in arterial blood, *PBW* = predicted body weight, *PEEP* = positive end-expiratory pressure, *SD* = standard deviation

^*^p < 0.05 when compared with patients complete renal recovery

Patients with late onset AKI (> 2 days after ARDS diagnosis) had lower severity of illness at the beginning of ARDS. However, they received more rescue therapies (54.3% vs 32.9%, *p* = 0.002), had a higher lactate (1.6 [1.1–2.3] vs 1.4 [1.0–1.7], *p* = 0.04) and a lower platelet count (163 [71–241] vs 221 [94–325], *p* = 0.02) on day 7 of ARDS.

### Renal recovery pattern according to severity of AKI

**Fig. 2:**
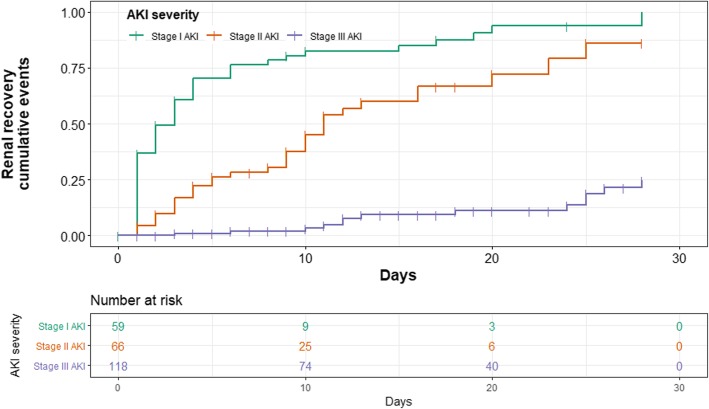
The graph shows cumulative events of renal recovery by staging of acute kidney injury in 243 patients

**Fig. 3:**
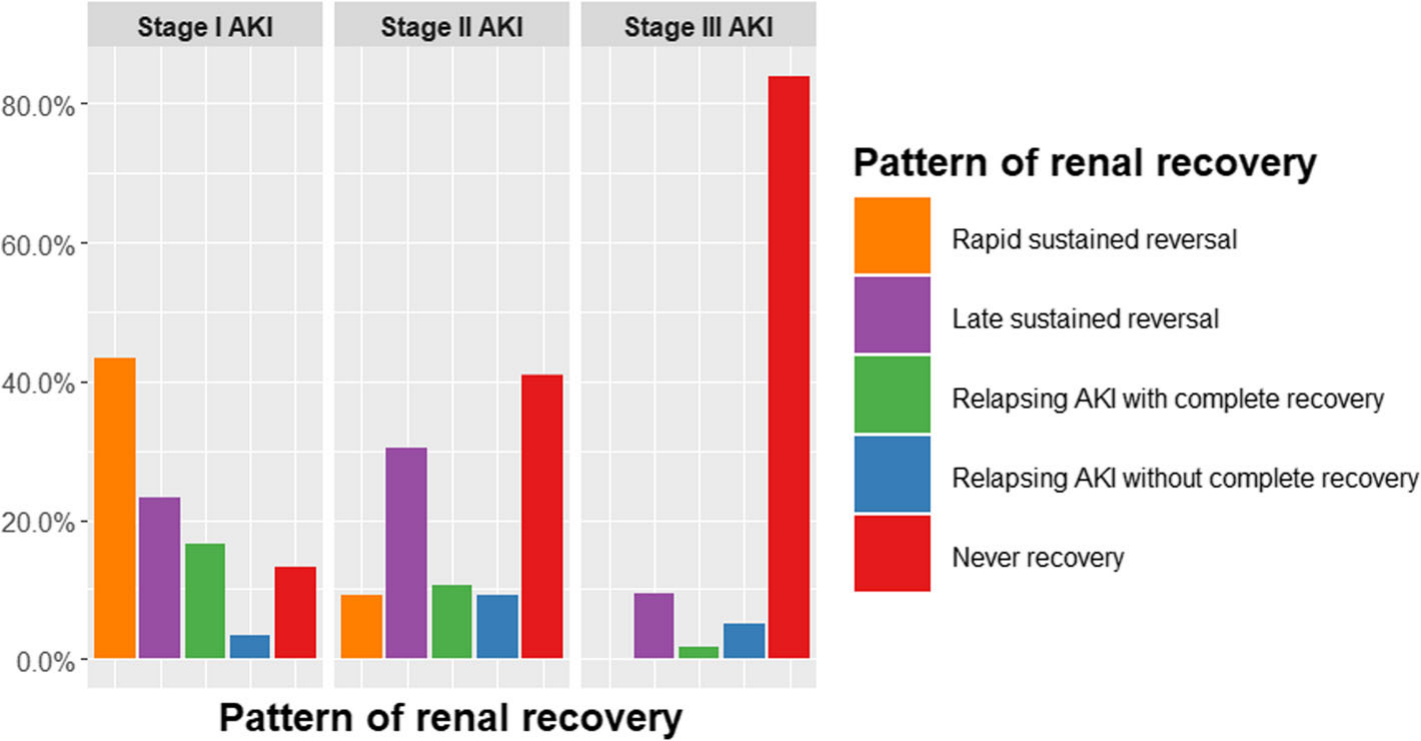
The bar graphs show pattern of renal recovery by staging of acute kidney injury in 244 patients. The patients with never recovery who died at day 28 were 91 (68%) patients

The rate of non-recovery increased with AKI severity, also the rate of complete renal recovery decreased from stage I to stage III AKI. Cumulative events of complete renal recovery were categorized by severity of AKI were shown in Fig.2. Among the 244 patients who developed AKI, 50 (83%) with stage I had complete renal recovery. Of those, 43% had a rapid recovery, and only 13% never recovered. Half of the patients with stage II had complete renal recovery. In this group late sustained reversal (30%) was observed to be more prevalent than rapid sustained reversal (9%). In contrast, 89% of patients with stage III did not have renal recovery at 28 days after AKI diagnosis or at hospital discharge (Table 3 and Fig. 3).

**Table 3:** Patterns of acute kidney injury reversal in acute respiratory distress syndrome

	Stage I AKI (60)	Stage II AKI (66)	Stage III AKI (118)
Complete renal recovery (%)	50 (83.3)	33 (50)	13 (11)
Non-renal recovery (%)	10 (16.7)	33 (50)	105 (89)
Rapid sustained reversal (%)	26 (43.3)	6 (9.1)	0 (0)
Late sustained reversal (%)	14 (23.3)	20 (30.3)	11 (9.3)
Relapsing AKI with complete recovery (%)	10 (16.7)	7 (10.6)	2 (1.7)
Relapsing AKI without complete recovery (%)	2 (3.3)	6 (9.1)	6 (5.1)
Never recovery (%)	8 (13.3)	27 (40.9)	99 (83.9)

*AKI* = acute kidney injury

We performed a sub-group analysis in only survivors, baseline characteristics and ventilator parameters are reported in Additional file 1: Tables S2 and S3 respectively. 40 (97.6%) patients with stage I AKI had complete renal recovery. Most of them had rapid sustained reversal (23; 56.1%) and late sustained reversal (9; 22%). The rate of renal non-recovery was higher with more severe AKI stages, 20.6% in patients with stage II and 75.5% in patients with stage III never recovered (see Additional file 1: Table S4 and Figure S1).

**Table 4:** Factors associated with renal non-recovery in patients with acute respiratory distress syndrome

Variable	Univariable analysis^a^			Multivariable analysis^b^		
	OR	95% CI	*p* value	OR	95% CI	*p* value
Severity of acute kidney injury						
Stage I	Ref	Ref	Ref	Ref	Ref	Ref
Stage II	5.00	2.24 to 11.98	< 0.001	5.71	2.17 to 14.98	< 0.001
Stage III	40.38	17.31 to 103.73	< 0.001	45.85	16.27 to 129.2	< 0.001
Acute kidney injury onset, day^c^	1.08	1.02 to 1.16	0.01	1.12	1.03 to 1.21	0.01
SOFA score^c^	1.20	1.11 to 1.31	< 0.001	–	–	–
History of heart failure	0.33	0.13 to 0.82	0.02	–	–	–
History of active malignancies	3.26	1.67 to 6.79	< 0.001	4.02	1.59 to 10.15	0.003
Septic shock	3.61	2.12 to 6.24	< 0.001	3.20	1.52 to 6.76	0.002
Mean tidal volume on day 1–3, mL/kg PBW^c^	1.31	1.08 to 1.61	0.01	1.41	1.05 to 1.90	0.02
Mean PEEP on day 1–3, cm H_2_O^c^	1.06	0.99 to 1.14	0.09	–	–	–
Continuous neuromuscular blocking agents	2.42	1.41 to 4.24	0.002	–	–	–
Inhaled vasodilators	1.95	1.07 to 3.7	0.03	–	–	–
Recruitment maneuvers	4.02	1.31 to 17.58	0.03	–	–	–
Antipsychotic drugs	0.61	0.36 to 1.02	0.06	–	–	–
Furosemide on day 2–7	0.48	0.28 to 0.82	0.01	–	–	–
Fluid overload on day 7, %^c^	1.07	1.02 to 1.12	0.005	–	–	–

*CI* = confidence interval, *OR* = odds ratio, *PBW* = predicted body weight, *Ref* = reference

Area under the receiver operating characteristic curve (95% CI) of multivariable analysis = 0.90 (95% CI 0.85–0.94)

^a^analysis from non-imputed data

^b^Pool analysis after multivariable logistic regression of 50 imputed data set

^c^per 1 point increase

**Table 5:** Factors associated with renal non-recovery in survival patients with acute respiratory distress syndrome

Variable	Univariable analysis^a^			Multivariable analysis^b^		
	OR	95% CI	*p* value	OR	95% CI	*p* value
Severity of acute kidney injury						
Stage I	Ref	Ref	Ref	Ref	Ref	Ref
Stage II	10.37	1.71 to 199.68	0.03	8.17	0.84 to 79.91	0.07
Stage III	123.08	23.18 to 2292.07	< 0.001	111.67	12.69 to 982.91	< 0.001
Acute kidney injury onset, day^c^	1.11	1.04 to 1.20	0.003	1.12	1.02 to 1.23	0.02
SOFA score^c^	1.19	1.05 to 1.35	0.01	–	–	–
History of active malignancies	3.87	1.46 to 11.12	0.01	6.55	1.34 to 32.04	0.02
Septic shock	1.97	0.96 to 4.11	0.07	–	–	–
Continuous neuromuscular blocking agents	1.93	0.91 to 4.10	0.09	–	–	–
Recruitment maneuvers	6.66	1.53 to 46.08	0.02	–	–	–
Antipsychotic drugs	2.09	0.99 to 4.57	0.06	–	–	–

*CI* = confidence interval, *OR* = odds ratio, *Ref* = reference

Area under the receiver operating characteristic curve (95% CI) of multivariable analysis = 0.90 (95% CI 0.84–0.95)

^a^analysis from non-imputed data

^b^Pool analysis after multivariable logistic regression of 50 imputed data set

^c^per 1 point increase

### Predictors of renal non-recovery

Fourteen potential variables were studied by multivariable analysis. Predictors associated with non-recovery in multivariable model are shown in Table 4. The severity of AKI was a strong predictor of non-recovery. Patients with stage II (odds ratio [OR] 5.71, 95% confidence interval [CI] 2.17–14.98, *p* < 0.001) and stage III (OR 45.85, 95% CI 16.27–129.2, *p* < 0.001) had significantly higher rates on non-recovery when compared to patients with stage I. Non-recovery was significantly associated with delayed onset of AKI (OR 1.12, 95%CI 1.03–1.21, *p* = 0.01), active malignancy (OR 4.02, 95% CI 1.59–10.15, *p* = 0.003) and septic shock (OR 3.2, 95% CI 1.52–6.76, *p* = 0.002). Patients who received a higher tidal volume on day 1–3 of ARDS had a significantly higher risk of non-recovery (OR 1.41, 95% CI 1.05–1.90, *p* = 0.02). Area under the receiver operating characteristic of multivariable model was 0.90 (95% CI 0.85–0.94). Subgroup analysis of patients who were alive at day 28 of ARDS also found that higher severity of AKI, delayed onset of AKI, and active malignancy were significant predictors of renal non-recovery (Table 5). The sensitivity analyses of predictors of renal non-recovery in non-imputed data are shown in Additional file 1: Table S5 and S6. These results were similar to the imputed data.

Since all patients who received RRT had stage III AKI by definition, we separately analyzed the effect of RRT on renal non-recovery only in patients with stage III. We found that patients with RRT had a higher likelihood of renal non-recovery (OR 3.65, 95% CI 1.13–12.9, *p* = 0.03). Effect of RRT on renal non-recovery was still significant in stage III patients who were alive at day 28. (OR 4.8, 95%CI 1.31–19.34, *p* = 0.02).

### Time to complete renal recovery

Among 96 patients with complete renal recovery, median time from AKI diagnosis to complete renal recovery increased with AKI severity. Patients with stage I had 2 days (IQR 1–4) while patients with stage II had 8 days (IQR 3–11), and patients with stage III had 13 days (IQR 11–25).

## Discussion

Our study found that in ARDS patients with AKI, a higher severity of initial AKI, delayed onset of AKI, active malignancy, septic shock, and a higher tidal volume on day 1 to 3 were associated with increased likelihood of renal non-recovery. Severity of AKI has been associated with a lower likelihood of renal recovery in critically ill patients [20–25]. Our study holds true for patients with ARDS, and worsening severity of AKI is associated with significantly higher chances of renal non-recovery. Delayed onset of AKI was also associated with renal non-recovery in our cohort. Patients with a delayed onset of AKI had a lower severity of illness on the first day of ARDS, the development of AKI was associated with a higher serum lactate and a lower platelet count over the course of their ICU stay. It is likely that patients with delayed onset AKI had a more complex hospital course with subsequent hemodynamic deterioration acquired during the course of ARDS. Patients who developed AKI after admission, showed a lower likelihood of renal recovery at hospital discharge [26].

Hypertension, cardiac disease, diabetes mellitus, and malignancy have been associated with renal non-recovery in critically ill patients [6, 25, 27, 28]. In our cohort of ARDS patients, active malignancy was associated with a higher chance of renal non-recovery. This finding has significant implications for clinical decision-making in this group of extremely ill patients.

Septic shock was associated with the risk of renal non-recovery in our study. The current literature has conflicting reports on the impact of sepsis on renal non-recovery [6, 24, 29, 30]. However, the severity and duration of AKI likely depend on the duration of the hemodynamic instability, underlying renal reserve, early treatment of sepsis, and timing of resuscitation [31]. These inconsistent findings may in part be due to different definitions of renal recovery and variability in the treatment of sepsis (hemodynamic optimization and type of fluid therapy) among the studies. Since AKI patients who died before day 28 of ARDS were classified in renal non-recovery and septic shock patients having a higher 28-day mortality, we restricted our analysis to survivors only. Among survivors, septic shock patients were not significantly associated with the risk of renal non-recovery.

Use of lower tidal volumes over the first three days of ARDS was significantly associated with renal recovery. This association has never been reported in published literature. But in experimental models it has been postulated that the deterioration in kidney function in ARDS might be a result of hemodynamic, neurohormonal, and biotrauma due to the use of higher tidal volumes [32, 33]. Thus use of lower tidal volumes can have a protective effect on recovery in these patients. Similar to the data presented in the LUNG SAFE study [13], almost 35% of our patients had a plateau pressure (Pplat) > 30 mm of H_2_0. Clinicians need to be mindful of adjusting ventilator settings more aggressively to maintain low inflation pressures and ensure that these patients are maintained a Pplat less than 30.

As reported in previous studies in critically ill patients initiation of RRT was associated with renal non-recovery in patients with ARDS [21, 22, 34]. The timing of RRT did not have an association with renal recovery. There is no consensus on the impact of timing of RRT in the current literature [35–37]. The difference in outcomes might be due to a difference in definitions of recovery used in these studies and the impact of unmeasured confounding factors [30, 38–40].

Several studies have described factors associated with renal recovery in critically ill patients, but this is the first study to explore this question in ARDS patients. We developed a very exhaustive model to account for any potential confounding from underlying comorbidities and ICU specific therapies. Also our study used the consensus renal recovery definition taking into account the SCr and urine output. We also excluded the patients who had CKD or AKI prior ARDS because CKD patients have a reduced renal function reserve, so it would affect the rate of renal recovery. However, this study also has some limitations. Transferred patients had some missing information in the first few days of ARDS values were averaged over the first 72 h of their ICU stay. We also performed a multiple imputation method to address any missing data. Schetz et al. found that recovery patterns in patients without CKD did not differ between true baseline SCr group and calculated baseline SCr group [22]. So for patients who did not have a baseline SCr, we estimated SCr by back calculation using the MDRD equation.

## Conclusions

Renal non-recovery is strongly associated with a higher severity of AKI, delayed onset of AKI, active malignancy, septic shock, and exposure to higher tidal volumes. However, renal non-recovery in survival patients was associated with a higher severity of AKI, delayed onset of AKI, and active malignancy.

## Additional file

Additional file 1: **Table S1.** Percentage of missing data of potential full model variables. **Table S2.** Baseline characteristics of survivors by renal recovery. **Table S3.** Ventilator settings, arterial blood gases averaged on day 1–3 and other therapies in survivors**. Table S4.** Patterns of acute kidney injury reversal in survivors with acute respiratory distress syndrome. **Table S5.** Factors associated with renal non-recovery in all patients (non-imputed data. **Table S6.** Factors associated with renal non-recovery in survival patients (non-imputed data. **Figure S1.** The bar graphs show pattern of renal recovery by staging of acute kidney injury in 128 survivors. (DOC 170 kb)

## Data Availability

The datasets used and/or analyzed during the current study are available from the corresponding author on reasonable request.

## Abbreviations

AKI: Acute kidney injury
APACHE: Acute Physiology, Age, Chronic Health Evaluation
ARDS: Acute respiratory distress syndrome
BMI: Body mass index
CI: Confidence interval
CKD: Chronic kidney disease
eGFR: estimated glomerular filtration rate
ICU: Intensive care unit
IQR: Interquartile range
KDIGO: the Kidney Disease Improving Global Outcomes
MDRD: Modification of Diet in Renal Disease
OR: Odds ratio
RRT: Renal replacement therapy
SCr: Serum creatinine
SOFA: Sequential Organ Failure Assessment

## References

[1] Cheungpasitporn W, Kashani K (2016). Electronic data systems and acute kidney injury. Contrib Nephrol.

[2] Srisawat N, Sileanu FE, Murugan R, Bellomod R, Calzavacca P, Cartin-Ceba R, Cruz D, Finn J, Hoste EE, Kashani K (2015). Variation in risk and mortality of acute kidney injury in critically ill patients: a multicenter study. Am J Nephrol.

[3] Darmon M, Clec'h C, Adrie C, Argaud L, Allaouchiche B, Azoulay E, Bouadma L, Garrouste-Orgeas M, Haouache H, Schwebel C (2014). Acute respiratory distress syndrome and risk of AKI among critically ill patients. Clin J Am Soc Nephrol.

[4] Forni LG, Darmon M, Ostermann M, Oudemans-van Straaten HM, Pettila V, Prowle JR, Schetz M, Joannidis M (2017). Renal recovery after acute kidney injury. Intensive Care Med.

[5] Chawla LS, Bellomo R, Bihorac A, Goldstein SL, Siew ED, Bagshaw SM, Bittleman D, Cruz D, Endre Z, Fitzgerald RL (2017). Acute kidney disease and renal recovery: consensus report of the acute disease quality initiative (ADQI) 16 workgroup. Nat Rev Nephrol.

[6] Kellum JA, Sileanu FE, Bihorac A, Hoste EA, Chawla LS (2017). Recovery after acute kidney injury. Am J Respir Crit Care Med.

[7] Jia HM, Zheng Y, Huang LF, Xin X, Ma WL, Jiang YJ, Zheng X, Guo SY, Li WX (2018). Derivation and validation of plasma endostatin for predicting renal recovery from acute kidney injury: a prospective validation study. Crit Care.

[8] Ranieri VM, Rubenfeld GD, Thompson BT, Ferguson ND, Caldwell E, Fan E, Camporota L, Slutsky AS (2012). Acute respiratory distress syndrome: the Berlin definition. JAMA..

[9] Palevsky PM, Liu KD, Brophy PD, Chawla LS, Parikh CR, Thakar CV, Tolwani AJ, Waikar SS, Weisbord SD (2013). KDOQI US commentary on the 2012 KDIGO clinical practice guideline for acute kidney injury. Am J Kidney Dis.

[10] Kidney Disease: Improving Global Outcomes (KDIGO) Acute Kidney Injury Work Group (2012). KDIGO clinical practice guideline for acute kidney injury. Kidney Int Suppl.

[11] Pickering JW, Endre ZH (2010). Back-calculating baseline creatinine with MDRD misclassifies acute kidney injury in the intensive care unit. Clin J Am Soc Nephrol.

[12] Kellum JA, Chawla LS, Keener C, Singbartl K, Palevsky PM, Pike FL, Yealy DM, Huang DT, Angus DC (2016). The effects of alternative resuscitation strategies on acute kidney injury in patients with septic shock. Am J Respir Crit Care Med.

[13] Bellani G, Laffey JG, Pham T, Fan E, Brochard L, Esteban A, Gattinoni L, van Haren F, Larsson A, McAuley DF (2016). Epidemiology, patterns of care, and mortality for patients with acute respiratory distress syndrome in intensive care units in 50 countries. JAMA..

[14] Sutherland SM, Zappitelli M, Alexander SR, Chua AN, Brophy PD, Bunchman TE, Hackbarth R, Somers MJ, Baum M, Symons JM (2010). Fluid overload and mortality in children receiving continuous renal replacement therapy: the prospective pediatric continuous renal replacement therapy registry. Am J Kidney Dis.

[15] Lombardi R, Nin N, Penuelas O, Ferreiro A, Rios F, Marin MC, Raymondos K, Lorente JA, Koh Y, Hurtado J (2017). Acute kidney injury in mechanically ventilated patients: the risk factor profile depends on the timing of Aki onset. Shock..

[16] Singer M, Deutschman CS, Seymour CW, Shankar-Hari M, Annane D, Bauer M, Bellomo R, Bernard GR, Chiche JD, Coopersmith CM (2016). The third international consensus definitions for Sepsis and septic shock (Sepsis-3). JAMA..

[17] Harris PA, Taylor R, Thielke R, Payne J, Gonzalez N, Conde JG (2009). Research electronic data capture (REDCap)--a metadata-driven methodology and workflow process for providing translational research informatics support. J Biomed Inform.

[18] Moons KG, Donders RA, Stijnen T, Harrell FE (2006). Using the outcome for imputation of missing predictor values was preferred. J Clin Epidemiol.

[19] van Buuren S, Groothuis-Oudshoorn K (2011). Mice: multivariate imputation by chained equations in R. J Stat Softw.

[20] Ali T, Khan I, Simpson W, Prescott G, Townend J, Smith W, Macleod A (2007). Incidence and outcomes in acute kidney injury: a comprehensive population-based study. J Am Soc Nephrol.

[21] Bouchard J, Acharya A, Cerda J, Maccariello ER, Madarasu RC, Tolwani AJ, Liang X, Fu P, Liu ZH, Mehta RL (2015). A prospective international multicenter study of AKI in the intensive care unit. Clin J Am Soc Nephrol.

[22] Schetz M, Gunst J, De Vlieger G, Van den Berghe G (2015). Recovery from AKI in the critically ill: potential confounders in the evaluation. Intensive Care Med.

[23] Long TE, Sigurdsson MI, Sigurdsson GH, Indridason OS (2016). Improved long-term survival and renal recovery after acute kidney injury in hospitalized patients: a 20 year experience. Nephrology (Carlton).

[24] Federspiel CK, Itenov TS, Mehta K, Hsu RK, Bestle MH, Liu KD (2018). Duration of acute kidney injury in critically ill patients. Ann Intensive Care.

[25] Hessey E, Ali R, Dorais M, Morissette G, Pizzi M, Rink N, Jouvet P, Lacroix J, Phan V, Zappitelli M (2017). Renal function follow-up and renal recovery after acute kidney injury in critically ill children. Pediatr Crit Care Med.

[26] Wonnacott A, Meran S, Amphlett B, Talabani B, Phillips A (2014). Epidemiology and outcomes in community-acquired versus hospital-acquired AKI. Clin J Am Soc Nephrol.

[27] Srisawat N, Wen X, Lee M, Kong L, Elder M, Carter M, Unruh M, Finkel K, Vijayan A, Ramkumar M (2011). Urinary biomarkers and renal recovery in critically ill patients with renal support. Clin J Am Soc Nephrol.

[28] Heung M, Wolfgram DF, Kommareddi M, Hu Y, Song PX, Ojo AO (2012). Fluid overload at initiation of renal replacement therapy is associated with lack of renal recovery in patients with acute kidney injury. Nephrol Dial Transplant.

[29] Bagshaw SM, Uchino S, Bellomo R, Morimatsu H, Morgera S, Schetz M, Tan I, Bouman C, Macedo E, Gibney N (2007). Septic acute kidney injury in critically ill patients: clinical characteristics and outcomes. Clin J Am Soc Nephrol.

[30] Bonnassieux M, Duclos A, Schneider AG, Schmidt A, Benard S, Cancalon C, Joannes-Boyau O, Ichai C, Constantin JM, Lefrant JY (2018). Renal replacement therapy modality in the ICU and renal recovery at hospital discharge. Crit Care Med.

[31] Fiorentino M, Tohme FA, Wang S, Murugan R, Angus DC, Kellum JA (2018). Long-term survival in patients with septic acute kidney injury is strongly influenced by renal recovery. PLoS One.

[32] Husain-Syed F, Slutsky AS, Ronco C (2016). Lung-kidney cross-talk in the critically ill patient. Am J Respir Crit Care Med.

[33] Domenech P, Perez T, Saldarini A, Uad P, Musso CG (2017). Kidney-lung pathophysiological crosstalk: its characteristics and importance. Int Urol Nephrol.

[34] Clark EG, Bagshaw SM (2015). Unnecessary renal replacement therapy for acute kidney injury is harmful for renal recovery. Semin Dial.

[35] Zarbock A, Kellum JA, Schmidt C, Van Aken H, Wempe C, Pavenstadt H, Boanta A, Gerss J, Meersch M (2016). Effect of early vs delayed initiation of renal replacement therapy on mortality in critically ill patients with acute kidney injury: the ELAIN randomized clinical trial. JAMA..

[36] Gaudry S, Hajage D, Schortgen F, Martin-Lefevre L, Verney C, Pons B, Boulet E, Boyer A, Chevrel G, Lerolle N (2018). Timing of renal support and outcome of septic shock and acute respiratory distress syndrome. A post hoc analysis of the AKIKI randomized clinical trial. Am J Respir Crit Care Med.

[37] Barbar SD, Clere-Jehl R, Bourredjem A, Hernu R, Montini F, Bruyere R, Lebert C, Bohe J, Badie J, Eraldi JP (2018). Timing of renal-replacement therapy in patients with acute kidney injury and Sepsis. N Engl J Med.

[38] Wald R, Shariff SZ, Adhikari NK, Bagshaw SM, Burns KE, Friedrich JO, Garg AX, Harel Z, Kitchlu A, Ray JG (2014). The association between renal replacement therapy modality and long-term outcomes among critically ill adults with acute kidney injury: a retrospective cohort study*. Crit Care Med.

[39] Liang KV, Sileanu FE, Clermont G, Murugan R, Pike F, Palevsky PM, Kellum JA (2016). Modality of RRT and recovery of kidney function after AKI in patients surviving to hospital discharge. Clin J Am Soc Nephrol.

[40] Truche AS, Darmon M, Bailly S, Clec'h C, Dupuis C, Misset B, Azoulay E, Schwebel C, Bouadma L, Kallel H (2016). Continuous renal replacement therapy versus intermittent hemodialysis in intensive care patients: impact on mortality and renal recovery. Intensive Care Med.

