# Correction to: Clinical predictors of renal non-recovery in acute respiratory distress syndrome

**DOI:** 10.1186/s12882-019-1479-7

**Published:** 2019-07-30

**Authors:** Anupol Panitchote, Omar Mehkri, Andrei Hastings, Tarik Hanane, Sevag Demirjian, Heather Torbic, Eduardo Mireles-Cabodevila, Sudhir Krishnan, Abhijit Duggal

**Affiliations:** 1 Department of Critical Care, Respiratory Institute, Cleveland Clinic, Cleveland, OH USA; 2 Division of Critical Care Medicine, Department of Medicine, Faculty of Medicine, Khon Kaen University, Khon Kaen, Thailand; 3 Department of Nephrology, Cleveland Clinic, Cleveland, OH USA; 4 Department of Pharmacology, Cleveland Clinic, Cleveland, OH USA

**Correction to: BMC Nephrology (2019) 20:255**

**https://doi.org/10.1186/s12882-019-1439-2**

Following publication of the original article [1], the authors reported that one of the authors’ names was spelled incorrectly. In this Correction the incorrect and correct author name are shown. The original publication of this article has been corrected.

Originally the author name was published as:

- Andrei Hasting

The correct author name is:

- Andrei Hastings
