# Supplementary material for: Clinical predictors of renal non-recovery in acute respiratory distress syndrome
Source: BMC Nephrol. 2019 Jul 10;20:255. doi: 10.1186/s12882-019-1439-2 (PMC6617675; doi:10.1186/s12882-019-1439-2)
Supplement: Supplementary file 1 — Table S1. Percentage of missing data of potential full model variables. Table S2. Baseline characteristics of survivors by renal recovery. Table S3. Ventilator settings, arterial blood gases averaged on day 1–3 and other therapies in survivors. Table S4. Patterns of acute kidney injury reversal in survivors with acute respiratory distress syndrome. Table S5. Factors associated with renal non-recovery in all patients (non-imputed data. Table S6. Factors associated with renal non-recovery in survival patients (non-imputed data. Figure S1. The bar graphs show pattern of renal recovery by staging of acute kidney injury in 128 survivors. (DOC 170 kb) [file 12882_2019_1439_MOESM1_ESM.doc]

**Clinical predictors of renal non-recovery in acute respiratory distress syndrome**

**Table S1** Percentage of missing data of potential full model variables

**Table S2** Baseline characteristics of survivors by renal recovery

**Table S3** Ventilator settings, arterial blood gases averaged on day 1-3 and other therapies in survivors

**Table S4** Patterns of acute kidney injury reversal in survivors with acute respiratory distress syndrome

**Table S5** Factors associated with renal non-recovery in all patients (non-imputed data)

**Table S6** Factors associated with renal non-recovery in survival patients (non-imputed data)

**Figure S1** The bar graphs show pattern of renal recovery by staging of acute kidney injury in 128 survival patients

**Table S1** Percentage of missing data of potential full model variables

| Variable | Percent of missing data |
| --- | --- |
| Percentage of fluid overload on day 7 | 44.7 |
| Average tidal volume on day 1-3 | 7.4 |
| Average PEEP on day 1-3 | 6.7 |
| SOFA score | 2.5 |
| Time from ARDS onset to AKI | 0.4 |

ARDS = acute respiratory distress syndrome; PEEP = positive end expiratory pressure; SOFA =sequential organ failure assessment.

**Table S2 Baseline characteristics of survivors by renal recovery**

| Characteristics | Recovery (80) | | Non-recovery (48) | | *p* value | |
| --- | --- | --- | --- | --- | --- | --- |
| Age, median (IQR), years | 55 | (43-64) | 51 | (42.5-62) |  | 0.49 |
| Male sex, n (%) | 47 | (58.8) | 27 | (56.2) |  | 0.78 |
| Body mass index, median (IQR), kg/m^2^ | 31.1 | (24.6-38.6) | 33.2 | (25.6-41.2) |  | 0.29 |
| Race, n (%)  White  Black or African American | 56  23 | (70)  (28.7) | 31  12 | (64.6)  (25) |  | 0.52  0.64 |
| SOFA, mean (SD), points | 10.4 | (2.9) | 12 | (3.3) |  | 0.01* |
| Non-renal SOFA, mean (SD), points | 9.6 | (2.5) | 10.5 | (2.8) |  | 0.06 |
| APACHE III, mean (SD), points | 105 | (30) | 118 | (29) |  | 0.02* |
| Charlson comorbidities index, median (IQR), points | 3 | (1-5) | 3 | (1-4.3) |  | 0.99 |
| Severity of ARDS on day 1  Mild  Moderate  Severe | 11  34  26 | (15.5)  (47.9)  (36.6) | 8  16  19 | (18.6)  (37.2)  (44.2) |  | 0.54 |
| Comorbidities, n (%)  Chronic lung diseases  Diabetes  Active malignancies  Liver disease  Heart failure  Recent surgery within 3 months. | 28  23  7  6  12  3 | (35)  (28.7)  (8.8)  (7.5)  (15)  (3.8) | 17  17  13  5  5  2 | (35.4)  (35.4)  (27.1)  (10.4)  (10.4)  (4.2) |  | 0.96  0.43  0.01*  0.75  0.46  1.00 |
| Cause of ARDS, n (%)  Pneumonia  Aspiration  Non-pulmonary sepsis  Pancreatitis | 68  15  6  5 | (85)  (18.8)  (7.5)  (6.2) | 42  7  5  1 | (87.5)  (14.6)  (10.4)  (2.1) |  | 0.69  0.55  0.75  0.41 |
| Echocardiographic findings  Ejection fraction, median (IQR),%  RVSP, median (IQR), mm Hg | 57  40 | (55-62)  (32.5-49) | 57  45 | (53.5-65)  (33-54) |  | 0.77  0.35 |
| Vasopressors use | 51 | (63.7) | 43 | (89.6) |  | 0.001 |
| Septic shock | 30 | (37.5) | 26 | (54.2) |  | 0.07 |
| Nephrotoxic agents | 77 | (96.2) | 47 | (97.9) |  | 1.00 |
| Median time to develop AKI | 3 | (2-6.3) | 3.5 | (3-11) |  | 0.02* |
| RRT initiation from highest AKI onset | 0 | (0-0) | 1 | (0-3) |  | 0.05 |
| Known baseline SCr, n (%) | 43 | (53.8) | 26 | (54.2) |  | 0.96 |
| Baseline SCr, mean (SD), mg/dL | 0.85 | (0.23) | 0.84 | (0.14) |  | 0.74 |
| eGFR, median (IQR), mL/min per 1.73 m^2^ | 95.6 | (73.6-111.4) | 94.6 | (82.5-109.2) |  | 0.68 |

AKI = acute kidney injury; APACHE = acute physiology, age, chronic health evaluation; ARDS = acute respiratory distress syndrome; eGFR = estimated glomerular filtration rate; IQR = interquartile range; RRT = renal replacement therapy; RVSP = right ventricular systolic pressure; SCr = serum creatinine; SD = standard deviation; SOFA = sequential organ failure assessment.

*p < 0.05 when compared with patients with complete renal recovery

**Table S3 Ventilator settings, arterial blood gases averaged on day 1-3 and other therapies in survivors**

| Ventilator settings | Recovery (80) | | Non-recovery (48) | | *p* value | |
| --- | --- | --- | --- | --- | --- | --- |
| Spontaneous tidal volume, median (IQR), mL | 478 | (431-538) | 487 | (433-531) |  | 0.64 |
| Tidal volume, median (IQR), (mL/kg PBW) | 7.2 | (6.5-8) | 7.3 | (6.7-8.5) |  | 0.10 |
| PEEP, median (IQR), cm H_2_O | 10 | (7.8-13.3) | 11.8 | (8.7-14.3) |  | 0.31 |
| FiO_2_, median (IQR) | 0.7 | (0.53-0.8) | 0.67 | (0.57-0.86) |  | 0.89 |
| Plateau pressure, median (IQR), cm H_2_O | 27 | (22.7-35) | 27 | (25-30.7) |  | 0.95 |
| Plateau pressure > 30 cm H_2_O, n (%) | 19 | (38.8) | 8 | (27.6) |  | 0.32 |
| Driving pressure, median (IQR), cm H_2_O | 15 | (12-19) | 14.5 | (12.3-16) |  | 0.30 |
| Mean airway pressure, median (IQR), cm H_2_O | 18.7 | (14.7-22) | 18 | (14.5-21) |  | 0.64 |
| Peak airway pressure, mean (SD), cm H_2_O | 31.4 | (7) | 31.3 | (7) |  | 0.94 |
| Minute ventilation, median (IQR), L/min | 11 | (9.4-12.9) | 11.1 | (9.9-12.3) |  | 0.87 |
| Arterial blood gas |  |  |  |  |  |  |
| Arterial pH, median (IQR) | 7.36 | (7.3-7.41) | 7.36 | (7.29-7.38) |  | 0.40 |
| PaCO_2_, median (IQR), mm Hg | 42.7 | (38-49.5) | 42 | (35-48.5) |  | 0.37 |
| PaO_2_, median (IQR), mm Hg | 87.7 | (74.3-114.6) | 94.3 | (79.7-124.5) |  | 0.34 |
| PaO_2_:FiO_2_, median (IQR) | 142 | (105-178) | 173 | (104-197) |  | 0.14 |
| Oxygenation index, median (IQR) | 15.7 | (9.4-23.6) | 13.7 | (8.6-20.9) |  | 0.51 |
| Rescue therapies, n (%) |  |  |  |  |  |  |
| Continuous neuromuscular blocking agents | 23 | (28.7) | 21 | (43.8) |  | 0.08 |
| Inhaled vasodilators | 14 | (17.5) | 12 | (25) |  | 0.31 |
| Prone positioning | 13 | (16.2) | 5 | (10.4) |  | 0.36 |
| Extracorporeal membrane oxygenation | 2 | (2.5) | 2 | (4.2) |  | 0.63 |
| Recruitment maneuvers | 2 | (2.5) | 7 | (14.6) |  | 0.03* |
| High frequency oscillatory ventilation | 1 | (1.2) | 0 | (0) |  | 1.00 |
| Other therapies |  |  |  |  |  |  |
| Sedative drugs, n (%) | 63 | (78.8) | 43 | (89.6) |  | 0.12 |
| Analgesic drugs, n (%) | 57 | (71.2) | 39 | (81.2) |  | 0.21 |
| Antipsychotic drugs, n (%) | 43 | (53.8) | 34 | (70.8) |  | 0.06 |
| Furosemide on day 2-7, n (%) | 51 | (63.7) | 31 | (64.6) |  | 0.92 |
| Fluid overload on day 7, mean (SD), % | 5.2 | (8.6) | 8.1 | (8.2) |  | 0.11 |

FiO_2_ = fraction of inspired oxygen; IQR = interquartile range; PaCO_2_ = partial pressure of carbon dioxide in arterial blood; PaO_2_ = partial pressure of oxygen in arterial blood; PBW = predicted body weight; PEEP = positive end-expiratory pressure; SD = standard deviation.

*P < 0.05 when compared with patients complete renal recovery

**Table S4 Patterns of acute kidney injury reversal in survivors with acute respiratory distress syndrome**

|  | Stage I AKI (41) | | Stage II AKI (34) | | Stage III AKI (53) | |
| --- | --- | --- | --- | --- | --- | --- |
| Complete renal recovery (%) | 40 | (97.6) | 27 | (79.4) | 13 | (24.5) |
| Non-renal recovery (%) | 1 | (2.4) | 7 | (20.6) | 40 | (75.5) |
|  | Stage I AKI (41) | | Stage II AKI (34) | | Stage III AKI (53) | |
| Rapid sustained reversal (%)  Late sustained reversal (%)  Relapsing AKI with complete recovery (%)  Relapsing AKI without complete recovery (%)  Never recovery (%) | 23  9  8  0  1 | (56.1)  (22)  (19.5)  (0)  (2.4) | 2  18  7  2  5 | (5.9)  (52.9)  (20.6)  (5.9)  (14.7) | 0  11  2  3  37 | (0)  (20.8)  (3.8)  (5.7)  (69.8) |

AKI = acute kidney injury.

**Table S5** Factors associated with renal non-recovery in all patients (non-imputed data)

| Variable |  | Multivariable analysis | | |  | |
| --- | --- | --- | --- | --- | --- | --- |
|  | Adjusted odds ratio | 95% confidence interval | | | *p* value | |
| Severity of acute kidney injury |  |  | | |  | |
| Stage I | Reference | Reference | | | Reference | |
| Stage II | 6.10 | 2.28 | to | 18.24 | < | 0.001 |
| Stage III | 74.01 | 24.97 | to | 260.35 | < | 0.001 |
| Acute kidney injury onset (day)^a^ | 1.13 | 1.03 | to | 1.24 |  | 0.01 |
| History of active malignancies | 4.30 | 1.65 | to | 12.12 |  | 0.004 |
| Septic Shock | 4.02 | 1.80 | to | 9.31 | < | 0.001 |
| Mean tidal volume on day 1-3 (mL/kg PBW)**^a^** | 1.46 | 1.10 | to | 1.97 |  | 0.01 |

PBW = predicted body weight.

Area under the receiver operating characteristic curve (95%CI) = 0.91 (95%CI 0.88-0.95).

^a^per 1 point increase

**Table S6** Factors associated with renal non-recovery in survival patients (non-imputed data)

| Variable |  | Multivariable analysis | | |  | |
| --- | --- | --- | --- | --- | --- | --- |
|  | Adjusted odds ratio | 95% confidence interval | | | *p* value | |
| Severity of acute kidney injury |  |  | | |  | |
| Stage I | Reference | Reference | | | Reference | |
| Stage II | 8.17 | 1.17 | to | 167.2 |  | 0.07 |
| Stage III | 111.67 | 19.48 | to | 2177.75 | < | 0.001 |
| Acute kidney injury onset (day)^a^ | 1.12 | 1.02 | to | 1.24 |  | 0.02 |
| History of active malignancies | 6.55 | 1.51 | to | 37.27 |  | 0.02 |

Area under the receiver operating characteristic curve (95%CI) = 0.90 (95%CI 0.84-0.95).

^a^per 1 point increase

Figure S1


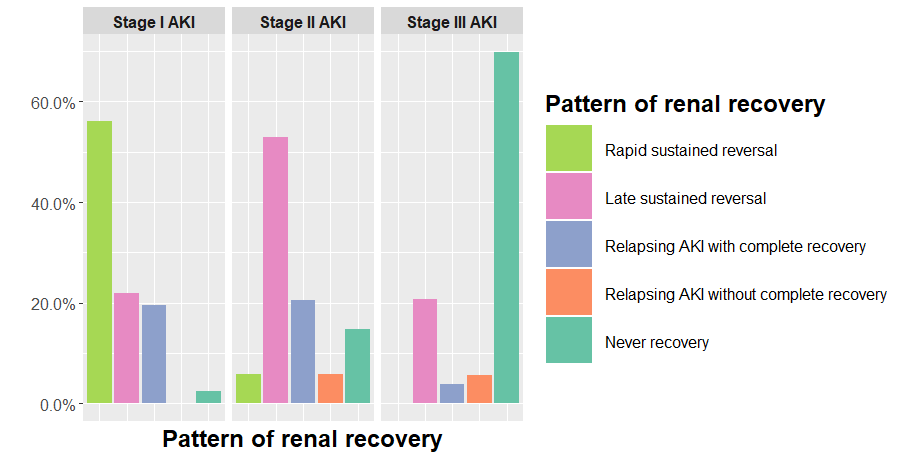


**Figure S2** The bar graphs show pattern of renal recovery by staging of acute kidney injury in 128 survivors.
